# ‘Am I Safe Here? Am I Going to Be Let Down?’ Disentangling Tensions in the Pursuit of Person‐Centred and Consumer Directed Care in the Australian Mental Healthcare Context

**DOI:** 10.1111/inm.70177

**Published:** 2025-11-21

**Authors:** Joshua McDonough, Mark Loughhead, Kate Rhodes, Monika Ferguson, Nicholas Procter

**Affiliations:** ^1^ Mental Health and Suicide Prevention Research and Education Group, Clinical and Health Sciences University of South Australia Adelaide South Australia Australia

**Keywords:** consumer directed care, healthcare systems, lived experience, mental health, person‐centred care, service integration

## Abstract

Healthcare concepts shape the way mental health care is conceived, delivered, experienced and evaluated. Person‐centred care and consumer‐directed care are two distinct but intertwined concepts that aim to redistribute knowledge and power between healthcare providers and consumers to ensure that healthcare is meeting the needs of consumers. However, despite many years of Australian services attempting to deliver person‐centred and consumer‐directed care, multiple reviews and inquiries into services find these attempts failing. The concepts of person‐centred and consumer‐directed care challenge the traditional ways in which mental health and mental health care have been conceived and delivered, reflecting tensions in the mental healthcare system. These tensions are theoretical, legislative and cultural. In this paper, the authors provide a description of these tensions, and highlight scenarios where these tensions have been overcome, and mental healthcare has been designed and delivered in a way that meets the needs of consumers. We provide ways forward that all stakeholders can implement to better our healthcare services, with a particular focus on mental health nurses.

## 1 Aims

The aim of this perspective article is to identify the common tensions within the Australian mental healthcare landscape that limit the successful integration of person‐centred and consumer‐directed care principles. We aim to identify the historical contexts and key drivers of these tensions and provide examples where they have been overcome and achieved good outcomes for consumers, carers, providers, and healthcare systems. In this paper, we attempt to provide a whole‐of‐system approach to the complexities of this transition, providing key ways forward for all to better implement these care concepts.

## 2 Background

I am seeking help with self‐harm and looking at “am I safe here? Is this another bad thing that's going to happen here? Am I going to be let down?” Consumer leader during the National Mental Health Commission consultation on person‐centred and consumer directed care (Loughhead et al. 2023)

More than ever, consumers, carers, families and kinship groups across Australia are demanding autonomy, choice and safety in mental health care responses. This includes deeper recognition that interaction and decision making in care is collaborative and participatory. Person‐centred care (PCC) is a concept that has formed over decades as modern health care practice has shifted. As such, it has been conceptualised in multiple ways, depending on the context of care (e.g., patient‐centred, person and family centred, woman‐centred, child‐centred, among others). While lacking a universally agreed upon definition, PCC has been defined as a healthcare approach that focuses on individuals, recognising unique needs, values and preferences over their diagnosis or medical condition(s) (Australian Commission on Safety and Quality in Health Care 2025). Key themes that define PCC in mental health are *autonomy* for individuals and adopting a *holistic* view of mental health (Loughhead et al. 2023). It is primarily driven by service providers to ensure that they can provide care to individuals that meets the needs of people at that specific timepoint. PCC involves including consumers, carers and kinship support in the decision‐making process to increase autonomy and choice for consumers and ownership of their own recovery outcomes.

Consumer directed care (CDC) has its origins in the disability and aged care sectors, born from a desire for independence and participation. It has been defined as a healthcare approach that gives individuals control over what care they receive, who provides it, as well as when and where it is provided (Gill et al. 2018). Key themes of CDC regarding mental health care include *choice* and *control* over care (Loughhead et al. 2023). It is primarily driven by the consumer to receive the care they want but is dependent on those services being available, accessible, usable and useful.

Despite being two distinct concepts, there are some overlapping principles between PCC and CDC. Both concepts take a strengths‐based approach, with goals to empower individuals to take an active role in choosing the types and frequency of the care they receive. Both concepts also express principles that overlap with recovery‐oriented practices, where individuals are empowered to live a meaningful and flourishing life, based on aspirations, goals and measures they have defined. As such, both concepts require relationships based on empathy and trust between consumers, carers and service providers to achieve success. Similarly, both concepts are built on the understanding that the mental health status of individuals can be influenced by social determinants (Kirkbride et al. 2024).

As the public health approach to mental health and suicide forms, there is attention being paid to the social determinants of mental health (Pirkis et al. 2024; Oswald et al. 2024). Understanding that mental ill health and suicidality are influenced by social determinants means recognising and acknowledging there are certain groups within society that experience poor mental health outcomes compared with the general public. In the context of suicide, there is a move to shift the narrative away from individual responsibility and mental health issues being the ‘cause’ of suicide, towards a holistic understanding of different drivers of suicidal thoughts and behaviours and how they can be addressed at a community level. This context of health inequalities places emphasis on supporting priority populations with improved levels of tailored and codesigned services. These have been noted in the Australian Government's recent Suicide Prevention Strategy (National Suicide Prevention Office 2025), and include people of migrant and refugee background, culturally and linguistically diverse communities, people in the LGBTQ+ community, and Aboriginal people, among others. Due to the unique needs and experiences of these communities, they often require specialist care responses, which depend on specific knowledges, workforces, support and referral pathways (Procter et al. 2023). As care frameworks, PCC and CDC present the opportunity to better define needs and preferred programmes of care with leaders and advisors from priority populations. Government agencies, care facilities, and care providers who are committed to PCC and CDC principles are therefore engaged in complex planning for simultaneously responding to multiple population and community group needs. This requires shifts away from generic models of the consumer towards co‐designed and flexible care models, relational skill sets and resourcing (Rock and Cross 2020).

Mental healthcare delivery in Australia is currently provided in a variety of ways, funded and delivered through a combination of government levels, insurers, private healthcare centres and individual providers. For example, the Federal Government funds consultations with specialist medical practitioners, general practitioners, psychologists and other allied health practitioners through the publicly funded universal healthcare scheme Medicare, while support for psychosocial disabilities is funded through the National Disability Insurance Scheme (NDIS). These services are primarily focused on early intervention and mental health recovery. State and territory governments fund and provide mental health services through public hospitals and emergency departments for people experiencing a mental health crisis, and residential and community mental health care for ongoing support and recovery. In addition to services provided or subsidised by governments, services are also provided by the community sector, such as crisis and support services like Lifeline and Beyond Blue. Services delivered in private hospitals are funded via private health insurance, contracted care through state and territory governments and/or out‐of‐pocket payments. Access to these services varies; among Australians experiencing ongoing mental health symptoms, 36% consulted a general practitioner, 21% a psychologist, 10% a psychiatrist and 13% another type of health professional in 2023 (Australian Institute of Health and Welfare 2024).

Within these different care delivery methods, PCC and CDC principles have long been accepted as standard practice. In Australia, both federal and state‐territory government directives cite these principles as key pillars of various services across the healthcare landscape. However, numerous reviews of mental health services within Australia have reported that experiences of person‐centred and consumer‐directed care are left wanting, including for Aboriginal children (Office of the Chief Psychiatrist 2024), for people with disability (Commonwealth of Australia 2023), for veterans (Commonwealth of Australia 2024), as well as services more generally (Victorian Government 2021). Many mental health consumers highlight that care responses are anything but person centred, with reports that consumers are often retraumatised by their experience of care (Hennessy et al. 2023). It is evident that despite commitments to these principles, there are barriers, tensions and cultural features within existing mental healthcare systems that inhibit the implementation of these principles into practice.

The aim of this paper is to extend our work in addressing and where possible disentangling these tensions, providing guidance to relevant stakeholders to better integrate PCC and CDC into the mental healthcare ecosystem. Mental health nurses are an integral part of the mental healthcare landscape. Their client and carer‐facing role coordinating therapeutic and recovery‐oriented practices provides an ideal opportunity to facilitate the delivery of person‐centred and consumer‐directed care. However, the barriers and tensions that inhibit the effective delivery of PCC and CDC extend beyond the responsibilities of mental health nurses. Therefore, the discussion of tensions in this paper has been kept broad to be relevant to lived experience, carers, healthcare providers, educators and policy makers within mental healthcare, with specific practice implications for mental health nurses included.

## 3 Design

The authors of this perspective piece recently completed a funded consultation process for the Australian National Mental Health Commission, bringing together 50 participants representing the perspectives of leaders in service provision, mental health policy, lived experience and peak bodies to outline how the concepts of PCC and CDC can be better incorporated into healthcare delivery (Loughhead et al. 2023). The final report was developed using a co‐design process involving a sub‐section of participants. The results of this work outline what outcomes can be expected from implementing PCC and CDC, what needs further discussion, and how we can measure their success. The conflicts and resolutions presented in this perspective piece are a combination of the work the authorship team did for the National Mental Health Commission, contemporary literature, as well as our collective experience working within the sector. Set in the Australian context, this paper was developed through ongoing discussions between the authorship team. The team includes a lived‐experience academic, mental health nurses, a psychologist and individuals with clinical and research expertise in mental health and suicide prevention.

## 4 Conflict 1: Models of Care

### 4.1 Tensions Between Biomedical vs. Bio‐Psycho‐Social Models of Care

Healthcare models affect how we conceptualise care, assess health and wellness, treat people and evaluate care outcomes. Adopting person‐centred and consumer‐directed principles into the Australian mental healthcare system inherently involves a continuing adaptation from biomedical to biopsychosocial models of care, focusing on a public health and social determinants approach to mental health and suicide prevention. Historical understandings of mental health were originally rooted in biomedical models, where clinicians diagnosed mental health ‘disorders’ based on deficiencies in neurological functioning (Handerer et al. 2021), and assumed biological illness processes. This way of thinking has framed how people providing mental health care perform their work. As such, for many years biological treatments using psychotropic medicines have been the predominant treatment for mental ‘disorders’. It is rare that people experiencing mental ill health present to healthcare services with a single issue, and often may seek support for a range of concerns, with a desire for multiple care initiatives focusing on a holistic view of health to have their needs met (Gask and Coventry 2012). Evidence suggests this approach to mental health care has failed consumers (Hennessy et al. 2023; Roennfeldt et al. 2024; McIntyre et al. 2024; Brophy et al. 2016; Hyde et al. 2015; Lim et al. 2024).

Mental health care informed by PCC and CDC principles recognises the biopsychosocial and lived experience context of consumers, enabling people to choose and adapt the care they receive. This would also include a holistic view of mental health, with various person‐reported outcomes important to them to be addressed (e.g., health‐related quality of life). This shift in healthcare models represents a tension, as it promotes a person's subjective assessments of health and wellness into care plans and emphasises that a person's mental health is felt and experienced, rather than a reflection of biological processes. This is in sharp contrast to other areas of healthcare, say cardiology, where there are biomarkers and medical imaging to assist clinicians with diagnosis and treatment success.

An example of shifting to person‐centred and consumer‐directed care principles in the model of care context is the implementation of Open Dialogue initiatives. Open Dialogue is being practiced in Australia, the United States and across Europe, with significant uptake in the United Kingdom. It aims to involve social and professional networks in discussions around care and treatment options and provide continuity of mental health care following a person's presentation to services. Open Dialogue prioritises community‐based treatment over hospital admission and seeks to address the power imbalance experienced by mental health service users, fostering the consumer's presence and autonomy in decision‐making (Freeman et al. 2019).

Studies evaluating Open Dialogue show reduced use of medicines, increased rates of reconnection with employment and less use of hospitals. However, current evaluation data for OD practice are weak, and generally difficult to consolidate (Freeman et al. 2019). A randomised trial is underway in the UK (Pilling et al. 2022), assessing the clinical outcomes and cost‐effectiveness of OD compared to conventional care response. As part of this project, a qualitative review of consumers' experiences 3 months after receiving peer‐supported Open Dialogue during a crisis showed that those who received OD felt they could rely on mental health services (Sunthararajah et al. 2022). They also reported valuing having choice and voice in their care, and the contributions made by their networks during clinical care. This is one example of how models of care can be shifted to change how we legislate and where we deliver mental health care.

## 5 Conflict 2: Legislative

### 5.1 Tensions Between Consumer Autonomy and Healthcare Provider Statutory Duties

The Australian mental healthcare system operates within a legislative framework that provides statutory and professional obligations for those who work within it. These laws outline the responsibilities of clinicians, and provisions that establish the conditions under which compulsory psychiatric intervention can occur. Being based on the biomedical model of mental health, this legislation grants clinicians, as the holders of knowledge and expertise, extraordinary powers to enforcedly detain a person under the relevant mental health act, allowing associated restrictive practices that significantly impact the autonomy of individuals through confinement, sedation and segregation (Patterson et al. 2016). This includes community treatment orders (CTOs), which require an individual to comply with treatment determined by a psychiatrist or authorised medical practitioner, including medication. The use of CTOs is increasing in Australia (Gill et al. 2020), indicating a strong focus on risk management while reflecting gaps in levels of care available for consumers (Harvery et al. 2025). Mental health legislation has an emphasis on risk and danger to oneself and others.

Along with the promotion of person‐centred and consumer‐directed care has been a focus on individual rights and autonomy in mental health. This is primarily outlined by the United Nations Convention on the Rights of Persons with Disabilities (CRPD), adopted in 2006 (United Nations 2006). This convention recognises that people experiencing mental distress, whether seeking treatment or not, have rights to autonomy and informed decision‐making over their care. As such, part of the convention calls for changes to legislative, administrative and other systems to preserve and support decision‐making rights of individuals, rather than substituted decision‐making by clinicians. Alongside a human rights approach, recovery model concepts also align with person‐centred and consumer‐directed approaches. While recovery is specific to an individual, common definitions include the ability to live a life of meaning within a chosen community, with or without the presence of mental health issues. Common values people associate with recovery‐oriented mental health care include self‐determination, empowerment, feelings of hope, transformation, discovery, connection, dignity and justice. Recovery is being increasingly conceptualised beyond being only a psychological process, but also a social and relational process (Price‐Robertson et al. 2017). This recognises that change occurs through relationships and opportunities that consumers choose, which results in healing and empowering experiences.

The inclusion of recovery as a mental healthcare principle elicits tensions with traditional legislative approaches to mental health care. This exists between recognising and supporting consumer autonomy and sense of safety, and statutory responsibilities to manage an individual's safety and risk, which can reinforce psychiatry's history of paternalism. Practitioners aiming to support recovery and positive risk taking in their work with consumers can face limits from risk‐averse workplace cultures, leading to fixed therapies and outcomes. Practitioners can also face a lack of strategic guidance from organisational leaders and policy settings, as well as limited local practice support to work through dilemmas and decisions (Holley et al. 2016; Harvery et al. 2025).

One approach to reduce legislative restrictions on autonomy and navigate recovery‐orientated practices within a legislative context has been advanced statements of person‐specific preferences. An advanced statement of preferences (sometimes called advanced care directives) can be completed by anyone, and it states individual preferences for treatment, care and support should they become subject to compulsory treatment, or a CTO (Department of Health Victoria 2025). These statements present the opportunity to empower individuals, enhance autonomy, promote early intervention, strengthen the therapeutic relationships between individuals and staff, and reduce compulsory admissions (Lasalvia et al. 2023). While evidence supports the use of advanced statements of preferences, their effectiveness can be reduced with unsupportive or inexperienced care providers, poor legal and ethical frameworks and poor communication.

These concepts have been further progressed through the SafeWards model (Department of Health Victoria 2022). This model aims to provide a safe and therapeutic environment for both consumers and staff in inpatient psychiatric services. It is a therapeutic approach inviting consumers to meet with mental health nurses in a group setting to discuss safety and preferred ward practices and routines. The aim is to generate safety and mutual support for consumers and nurses to reduce conflict, restrictive practices and coercive outcomes (Department of Health Victoria 2022).

Evaluations of the ‘Safewards’ model have produced encouraging outcomes, reducing the duration of coercive interventions and exposing fewer consumers to coercive interventions altogether (Baumgardt et al. 2019; Daniel et al. 2025). A systematic review of mixed‐methods studies on the efficacy of Safewards interventions suggests Safewards interventions are closely associated with reduced rates of conflict, reduced rates of containment practices, and increases in both consumers' and staff's perceived experience of safety in the ward (Ward‐Stockham et al. 2022). The review concludes that consumers and staff reported improved therapeutic relationships, cohesion and ward atmosphere, leading to consumer‐centred and recovery‐oriented care.

The practice of supported decision making, which should be embedded across all contexts of inpatient and community care, is an essential movement for promoting both autonomy and support. Analysis on successfully realising supported decision making as a cultural and practiced routine of mental health care requires much more than its presence in legislation; it needs embedding via new models of care, workforce skills and training, governance processes and regulation practices (Katterl and Maylea 2021).

## 6 Conflict 3: Workforce Culture

### 6.1 Tensions Between Cultures of Clinical Care and Recovery

The history of mental healthcare being rooted in the biomedical model has resulted in a healthcare landscape where power (and by extension, autonomy) is weighted heavily towards clinicians. The assumption that mental ill health is the result of neurological deficiencies lends itself to assuming that all knowledge of the condition and how to manage it is best placed with the clinician. Indeed, as discussed previously, mental health legislation can grant clinicians the option to remove a consumer's autonomy to force treatment upon an individual. This paternalistic view of healthcare can result in monologic coercive treatment through forced choices, medications and isolation, as well as—although rare—threats and violence. The inclusion of peer workers into multidisciplinary teams as recovery leaders has increased in recent years. Peer workers draw upon and utilise their lived experience to support another person through experiences of emotional distress, navigation of services and recovery goals. They also act as change agents in promoting shifts towards recovery language and service culture (Reeves, Loughhead, Teague, et al. 2024; McIntyre et al. 2024). The overarching goal of including the perspective of those with lived and living experience of mental distress is to understand drivers, mitigations, meanings and comforts for distress from their perspective. Such steps provide a more holistic approach to care plans, increasing consumer choice and autonomy, facilitating shared decision making and reestablishing a relational basis of healing and growth. These run counter to practices of objectification through assessment, an emphasis on pathology and risk, and distrust of consumer perspectives, which have been historically embedded in mental models and service culture. Examples of peer‐led models include Intentional Peer Support (Mead 2025), Emotional CPR, safe haven community‐based alternatives to emergency departments (Heyland and Johnson 2017), and peer‐led suicide prevention programmes (Wilson et al. 2022; Schlichthorst et al. 2020).

The inclusion of the peer workforce into mental health care teams has amplified tensions between the traditional ways of working, and a more holistic, emancipatory themed approach to recovery. These tensions are complex and multi‐faceted. For example, it has been argued that the inclusion of peer workers in mental healthcare forces working within pre‐existing biomedical models, which obstruct reform and critique of services (Sinclair et al. 2024), and discounts the change agent role of peer work. The inclusion of peer workers without broader meaningful reform can skew the scope of practice for peer workers through encouraging peer involvement in traditional practice, such as medication adherence. Alternatively, non‐peer workers may place unfair expectations on peer workers, expecting significant improvement in consumer outcomes in a short timeframe, and discounting peer work when those expectations are not met. Those approaching healthcare from a biomedical stance may view peer work at the bottom of the healthcare hierarchy, therefore discounting its value. The integration of these workforces will not be successful unless there are genuinely open and safe spaces for individuals to express their views and work together (Reeves, Loughhead, Halpin, and Procter 2024).

Successful integration of peer work within mental health services will require reform across and within services. This is challenging, as healthcare systems are complex, sometimes disjointed, and contain various levels of responsibility and ownership as part of a larger health system. These factors also exist within the context of healthcare systems needing to perform their primary objective on a 24‐h basis; providing healthcare. In addition to this systems approach, the needs and conditions of individuals must be considered. The mental healthcare workforce in Australia is under pressure, with staff shortages and challenging and unsupportive workplace environments leading to stressed and burnt‐out mental healthcare providers (The Department of Health and Aged Care 2023; McDonough et al. 2025). Services will need to address these stresses and appropriately resource and support the mental health workforce to shift culture, knowledge and practice to meet the objectives of PCC and CDC.

Healthcare services can be further supported using lived experience leadership. Lived experience leadership is about driving change from peer perspectives, transcending personal, organisational and public boundaries. While leaders are often advocates, researchers, peer workers, community educators and activists, it's the leadership action, guided by peer values and knowledge that is central (Loughhead et al. 2020). Lived experience leaders bring value to organisational operation and policy decision‐making. This includes critical insights and perspectives on justice issues, stigma and service preferences and advocacy goals (Day et al. 2017). Leaders also bring links with consumer networks, credibility and recovery values (Scholz et al. 2017).

There is scope to develop new services that are co‐designed from inception, where consumer and carer leaders work with practitioners, policy makers and the community to rethink and create new services, information resources and support programmes. These services can exist outside of traditional tertiary healthcare institutions and address some of the shortfalls of traditional mental health culture and treatment, including site‐based care, telehealth and community/residential care. Codesigned services can also be lived experience led and delivered. Indeed, this recommendation was included in the final report of the Royal Commission into Victoria's Mental Health System.

In the Australian context, an example of a codesigned new site‐based approach to mental healthcare includes the Federal Government funded *Medicare Mental Health Centres* (previously called *Head to Health* centres). This service is designed to provide support for people experiencing a mental health crisis where care is delivered through transdisciplinary teams of peer workers and clinicians (Department of Health, Disability, and Ageing 2021). Initial evaluation data of these centres is positive, with users appreciating a mix of peer and non‐peer workers, choice of supports and safe, non‐clinical environments (Neami National 2024). Nearly one third of service users are engaging with mental health support for the first time; the majority are self‐referred, and one quarter would not have sought healthcare elsewhere (Neami National 2024). This indicated that the inclusion of these centres into the mental healthcare landscape in Australia is having the desired effect of better reaching people experiencing mental distress and reducing mental health presentations to emergency departments. The latter point is particularly poignant in the Australian context, as cities and towns around the country experience high levels of ambulance ‘ramping’, a practice where people presenting to hospitals are kept in an ambulance because there is no available bed within the emergency department. People presenting to emergency departments for mental health‐related concerns disproportionately experience longer wait times and length of stay than non‐mental health presentations (Simko et al. 2022; Pascoe et al. 2022). They are also at risk of trauma through inappropriate care responses, discrimination and diagnostic overshadowing (McIntyre et al. 2024). Having more non‐hospital‐based options for people experiencing a mental health crisis can provide better care and reduce pressure on hospital emergency departments, creating an overall more efficient health system.

## 7 Conclusions

Growing numbers of reviews, evaluations and Royal Commissions into aspects of the Australian mental health care system provide more examples of mental health care failing to meet the needs of consumers, carers and their kin networks, even sometimes doing more harm than good. They tell stories of a workforce that is burnt out, understaffed and confined by funding constraints and narrow measures of success. This discursive paper has collated the experience of the authorship team and contemporary literature to outline some of the major tensions within the Australian mental healthcare landscape that arise during the transition and ongoing development of implementing person‐centred and consumer‐directed mental health care. We do not expect this to be a complete picture of the problems with implementing PCC and CDC in the Australian mental healthcare system. Mental health care is so dynamic and context‐specific that nearly each interaction will be unique. Instead, we aim to provide a clear path forward for each relevant stakeholder to work within and between organisations and practices to strengthen mental healthcare delivery.

Disentangling these tensions is a complex task. It involves system‐level reform and commitment across various sectors, including shifts in education, policy, government and healthcare providers. There are some practice models that are showing promise in relieving some of these tensions, including Open Dialogue approaches, the SafeWards model and peer‐designed and delivered crisis support centres. However, genuine PCC and CDC principles will only be fully implemented in the Australian mental healthcare sector with genuine support and nurturing from all relevant stakeholders at all levels. This will include unlearning ingrained practices, learning new ones, listening to people with lived and living experience and coming together in an open and honest way to build a resilient and effective mental healthcare system that delivers for consumers and workers equally.

## 8 Relevance for Practice

Mental health nurses have a vital role to play in the delivery of person‐centred and consumer‐directed care. This is achieved in their daily work, working side by side with consumers, carers, families, peer workers and kinship groups in Australia to promote autonomy, choice, and safety in mental health care responses. Being open to sharing power brings together compassion and caring and evidence‐informed practice at the point of care. Demonstrate understanding that mental ill health and suicidality are complex events and experiences influenced by social determinants; indeed many mental health nurses already do (Wand et al. 2022). For mental health nurses this means recognising and acknowledging there are certain groups and individuals within society that experience poor mental health outcomes compared with the wider public. For some mental health nurses this requires shifts away from generic models of the consumer towards co‐designed and flexible care models, relational skill sets and empowerment of peer workers for person‐centred and consumer‐directed care responses.

We have developed recommendations for changes and reforms that aim to provide guidance to each key stakeholder as to how they can instil, nourish and develop person‐centred and consumer‐directed care principles into the Australian mental healthcare system to address the aforementioned tensions (Table 1). Additionally, it provides points that consumers, carers, healthcare workers and policy makers can advocate for when calling for system reform.

**TABLE 1:** Recommendations for relevant stakeholders to guide the implementation of PCC and CDC principles into the Australian mental healthcare system.

Stakeholder	Actions
Universities	Embed critical review and discussion of PCC and CDC concepts and applications into relevant courses at undergraduate and postgraduate levels across all levels of health and service delivery. This can include multi‐disciplinary care and recovery orientated care principles, among others. This gives new workforce entrants across the mental healthcare landscape foundational understanding of the key principles and the role they play as care and service providers while transitioning to chosen careers. Fund and support lived‐experience academic positions to strengthen the voice of lived experience in research and teaching development and delivery.
Governments and government‐commissioned health services	Integrate and embed the use of supported decision‐making practices (e.g., mental health advanced care directives, nominated support people) in healthcare legislation and practice. Support staff to continually upskill and refresh knowledge and application practices of person‐centred and consumer directed care. This can be done through strong partnerships between government and universities to provide up‐to‐date, best practice training for healthcare workers. Strengthen practitioner and lived experience leadership within organisation governance and workforce to facilitate the championing of organisational and systems change. Promote crisis response models that emphasis consumer's dignity, personal safety and cultural safety. Fund long‐term care programmes with a ‘relational recovery’ focus, allowing for holistic care to respond to intersecting social determinants of mental health. Fund and support lived experience organisations to provide peer navigation services to assist consumers and carers with successful access to the health system.
Professional colleges	Work towards providing opportunities to strengthen practitioner identity, education and training throughout workers' career.
Peak bodies and non‐government organisations	Advocate for ongoing development, integration and evaluation of PCC and CDC concepts in healthcare settings.

## Author Contributions

All authors contributed to the conceptualisation of this manuscript and have reviewed the work. J.M. and M.L. prepared the manuscript for publishing.

## Funding

The initial stages of this article were from work funded by the Australian National Mental Health Commission.

## Conflicts of Interest

The authors declare no conflicts of interest.

## Data Availability

Data sharing not applicable to this article as no datasets were generated or analysed during the current study.
